# Active-Site Inhibitors of mTOR Target Rapamycin-Resistant Outputs of mTORC1 and mTORC2

**DOI:** 10.1371/journal.pbio.1000038

**Published:** 2009-02-10

**Authors:** Morris E Feldman, Beth Apsel, Aino Uotila, Robbie Loewith, Zachary A Knight, Davide Ruggero, Kevan M Shokat

**Affiliations:** 1 Howard Hughes Medical Institute and Department of Cellular and Molecular Pharmacology, University of California San Francisco, San Francisco, United States of America; 2 Department of Molecular Biology, University of Geneva, Switzerland; 3 School of Medicine and Department of Urology, Helen Diller Family Comprehensive Cancer Center, University of California San Francisco, San Francisco, United States of America; Salk Institute for Biological Studies, United States of America

## Abstract

The mammalian target of rapamycin (mTOR) regulates cell growth and survival by integrating nutrient and hormonal signals. These signaling functions are distributed between at least two distinct mTOR protein complexes: mTORC1 and mTORC2. mTORC1 is sensitive to the selective inhibitor rapamycin and activated by growth factor stimulation via the canonical phosphoinositide 3-kinase (PI3K)→Akt→mTOR pathway. Activated mTORC1 kinase up-regulates protein synthesis by phosphorylating key regulators of mRNA translation. By contrast, mTORC2 is resistant to rapamycin. Genetic studies have suggested that mTORC2 may phosphorylate Akt at S473, one of two phosphorylation sites required for Akt activation; this has been controversial, in part because RNA interference and gene knockouts produce distinct Akt phospho-isoforms. The central role of mTOR in controlling key cellular growth and survival pathways has sparked interest in discovering mTOR inhibitors that bind to the ATP site and therefore target both mTORC2 and mTORC1. We investigated mTOR signaling in cells and animals with two novel and specific mTOR kinase domain inhibitors (TORKinibs). Unlike rapamycin, these TORKinibs (PP242 and PP30) inhibit mTORC2, and we use them to show that pharmacological inhibition of mTOR blocks the phosphorylation of Akt at S473 and prevents its full activation. Furthermore, we show that TORKinibs inhibit proliferation of primary cells more completely than rapamycin. Surprisingly, we find that mTORC2 is not the basis for this enhanced activity, and we show that the TORKinib PP242 is a more effective mTORC1 inhibitor than rapamycin. Importantly, at the molecular level, PP242 inhibits cap-dependent translation under conditions in which rapamycin has no effect. Our findings identify new functional features of mTORC1 that are resistant to rapamycin but are effectively targeted by TORKinibs. These potent new pharmacological agents complement rapamycin in the study of mTOR and its role in normal physiology and human disease.

## Author Summary

Growth factor pathways are required for normal development but are often inappropriately activated in many cancers. One growth-factor–sensitive pathway of increasing interest to cancer researchers relies on the mammalian target of rapamycin (mTOR), a kinase that (like all kinases) delivers phosphate groups from ATP to amino acid residues of downstream proteins. TOR proteins were first discovered in yeast as the cellular targets of rapamycin, a small, naturally occurring molecule derived from bacteria that is widely used as an immunosuppressant and more recently in some cancer therapies. The study of TOR proteins has relied heavily on the use of rapamycin, but rapamycin does not directly inhibit TOR kinase activity; rather, rapamycin influences TOR's enzymatic activities by binding to a domain far from the kinase's active site. Some mTOR functions are resistant to rapamycin, as a result of the kinase activity of one kind of multiprotein complex, the mTOR complex 2 (mTORC2), whereas rapamycin-sensitive functions of mTOR are due to the mTOR complex 1 (mTORC1). We have developed new inhibitors of mTOR that bind to the ATP-binding site of mTOR and inhibit the catalytic activity of both mTORC1 and mTORC2 without inhibiting other kinases. Unexpectedly, these inhibitors had profound effects on protein synthesis and cell proliferation due to their inhibition of mTORC1 rather than mTORC2. We found that the phosphorylation of a protein that controls protein synthesis, the mTORC1 substrate 4E binding protein (4EBP) is partially resistant to rapamycin but fully inhibited by our new inhibitors. The finding that 4EBP phosphorylation is resistant to rapamycin suggests that active-site inhibitors may be more effective than rapamycin in the treatment of cancer and may explain why rapamycin is so well tolerated when taken for immunosuppression.

## Abstract 3

Cells rely on the mammalian target of rapamycin kinase (mTOR) to sense growth factors. Inhibition of all forms of mTOR using newly developed inhibitors of its active site reveals new insights into the function of two mTOR-containing protein complexes and their potential as therapeutic targets.

## Introduction

The mammalian target of rapamycin (mTOR) is a serine-threonine kinase related to the lipid kinases of the phosphoinositide 3-kinase (PI3K) family. mTOR exists in two complexes, mTORC1 [1,2] and mTORC2 [3,4], which are differentially regulated, have distinct substrate specificities, and are differentially sensitive to rapamycin. mTORC1 integrates signals from growth factor receptors with cellular nutritional status and controls the level of cap-dependent mRNA translation by modulating the activity of key translational components such as the cap-binding protein and oncogene eIF4E [5].

mTORC2 is insensitive to rapamycin, and selective inhibitors of this complex have not been described. Partly because acute pharmacological inhibition of mTORC2 has not been possible, the functions of mTORC2 are less well understood than those of mTORC1. mTORC2 is thought to modulate growth factor signaling by phosphorylating the C-terminal hydrophobic motif of some AGC kinases such as Akt [3,6] and SGK [7] although other kinases, including DNA-PK and Ilk, have also been implicated in Akt hydrophobic motif phosphorylation [8–11]. Growth factor stimulation of PI3K causes activation of Akt by phosphorylation at two key sites: the activation loop (T308) and the C-terminal hydrophobic motif (S473). Active Akt promotes cell survival in many ways, including suppressing apoptosis, promoting glucose uptake, and modifying cellular metabolism [12]; consequently, there is significant interest in identifying the kinase(s) responsible for each activating phosphorylation, the relationship between these phosphorylation sites, and the role of differential Akt phosphorylation on Akt substrate phosphorylation. Of the two phosphorylation sites on Akt, activation loop phosphorylation at T308, which is mediated by PDK1, is indispensable for kinase activity, whereas hydrophobic motif phosphorylation at S473 enhances Akt kinase activity by approximately 5-fold [13].

The disruption of mTORC2 by different genetic and pharmacological approaches has variable effects on Akt phosphorylation. Targeting mTORC2 by RNA interference (RNAi) [6,14], homologous recombination [15–17], or long-term rapamycin treatment [18] results in loss of Akt hydrophobic motif phosphorylation (S473), strongly implicating mTORC2 as the kinase responsible for phosphorylation of this site. RNAi targeting mTORC2 and long-term rapamycin result in loss of Akt phosphorylation on its activation loop (T308), but this phosphorylation remains intact in mouse embryonic fibroblasts (MEFs) lacking the critical mTORC2 component SIN1. It cannot be inferred from this genetic data whether acute pharmacological inhibition of mTORC2 would block the phosphorylation of Akt only at S473, resulting in partial Akt deactivation, or also disrupt phosphorylation at T308, resulting complete Akt inhibition.

Several small molecules have been identified that directly inhibit mTOR by targeting the ATP binding site; these include LY294002, PI-103, and NVP-BEZ235 [19–22]. These molecules were originally discovered as inhibitors of PI3Ks and later shown to also target mTOR. Because all of these molecules inhibit PI3Ks and mTOR with similar potency, they cannot be used to selectively inhibit mTOR or PI3Ks in cells. Indeed, because mTORC1 and mTORC2 function downstream of PI3Ks in most settings, it is unclear to what extent the ability of these molecules to block the activation of signaling proteins such as Akt reflects PI3K versus mTOR inhibition. It is possible that some of the functions attributed to PI3Ks using the classical inhibitor LY294002 are a consequence of mTOR inhibition [19,23], but it is has not been possible address this, because small molecules that inhibit mTOR without inhibiting PI3Ks have not been available.

We recently reported the synthesis of pyrazolopyrimidines that inhibit members of the PI3K family, including mTOR [24]. Two of these molecules, PP242 and PP30, are the first potent, selective, and ATP-competitive inhibitors of mTOR. Unlike rapamycin, these molecules inhibit both mTORC1 and mTORC2, and, unlike PI3K family inhibitors such as LY294002, these molecules inhibit mTOR with a high degree of selectivity relative to PI3Ks and protein kinases. To distinguish these molecules from the allosteric mTORC1 inhibitor rapamycin, we are calling them “TORKinibs” for TOR kinase domain inhibitors. The dual role of mTOR within the PI3K→Akt→mTOR pathway as both an upstream activator of Akt and the downstream effector of pathway activity on cell growth and proliferation has excited interest in active-site inhibitors of mTOR [25–30]. We describe here the biological activity of these molecules.

Another small-molecule ATP-competitive mTOR inhibitor called Torin1 was reported while our manuscript was in the process of publication [56].

## Results

### Specific Active-Site Inhibition of mTOR by the TORKinibs PP242 and PP30

**Figure 1:**
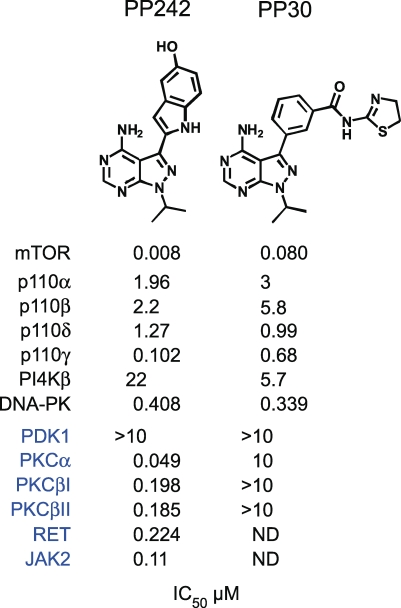
In Vitro IC_50_ Values for PP242 and PP30 Determined in the Presence of 10 μM ATP

PP242 and PP30 inhibit mTOR in vitro with half-maximal inhibitory concentrations (IC_50_ values) of 8 nM and 80 nM, respectively. As expected for active-site inhibitors, PP242 and PP30 inhibit mTOR in both mTORC1 and mTORC2 (Table S1). Both compounds are selective within the PI3K family, inhibiting other PI3Ks only at substantially higher concentrations (Figure 1). Testing of PP242 against 219 purified protein kinases at a concentration 100-fold higher than its mTOR IC_50_ value revealed exceptional selectivity with respect to the protein kinome; most protein kinases were unaffected by this drug, and only four—PKC-alpha, PKC-beta, RET, and JAK2 (V617F)—were inhibited more than 80% [24]. We determined IC_50_ values for PP242 against these kinases in vitro using purified proteins. In these assays, PP242 was relatively inactive against PKC-beta, RET, or JAK2 but inhibited PKC-alpha with an in vitro IC_50_ of 50 nM (Figure 1). Importantly, PP30 showed no activity against PKC-alpha or PKC-beta in the same assay (Figure 1). These data indicate that PP242 is a highly selective inhibitor of mTOR and that PP30 can be used to confirm that the effects of PP242 are due to inhibition of mTOR and not PKC-alpha. The availability of a second structurally dissimilar mTOR inhibitor—PP30—provides additional control for unanticipated off-targets of PP242.

### Inhibition of mTORC2 and Akt Phosphorylation by TORKinibs

**Figure 2:**
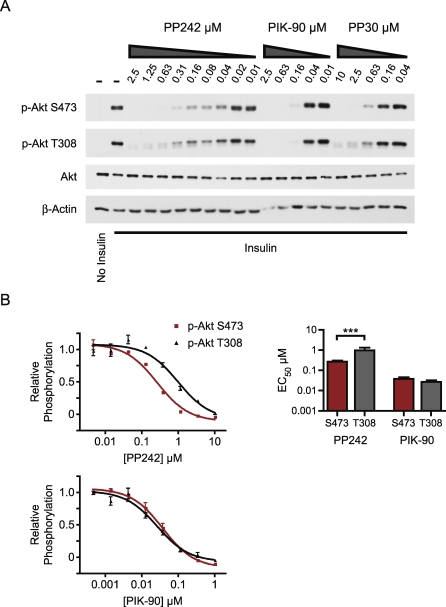
Inhibition of mTORC2 by TORKinibs Affects pS473 and pT308 of Akt (A) Serum-starved L6 myotubes were pre-treated with kinase inhibitors prior to stimulation with insulin for 3 min. Lysates were analyzed by Western blotting. (B) PP242 inhibits pS473 (red) of Akt more potently than pT308 (gray). Serum-starved L6 myotubes were treated with kinase inhibitors prior to stimulation with insulin for 10 min. Akt phosphorylation was measured by in-cell Western and is shown relative to serum starvation and insulin stimulation (*n* = 3 for each inhibitor dose). EC_50_ values from the best fit curves are plotted. ***p < 0.001, F test. EC_50_ values for PIK-90 on pS473 and pT308 were not significantly different (*p* = 0.2, F test).

We characterized the effect of PP242 on the PI3K→Akt→mTOR pathway. PP242 and PP30 both inhibited insulin-stimulated phosphorylation of Akt at S473, confirming that mTOR kinase activity is required for hydrophobic motif phosphorylation (Figure 2A). The inhibition of mTOR by PP242 and PP30 also resulted in loss of Akt phosphorylation at T308, but significantly higher doses of PP242 and PP30 were required to inhibit T308 as compared with S473 (Figure 2A and 2B). PP242 inhibited S473-P and T308-P at both early and late time points after insulin stimulation, indicating that the differential sensitivity of these sites to PP242 does not reflect differing kinetics of phosphorylation (Figure S1). By comparison, the PI3K inhibitor PIK-90, which does not inhibit mTOR, inhibited the phosphorylation of both Akt sites equipotently (Figure 2B), as observed previously [21].

We sought to confirm that the loss of T308-P caused by PP242 and PP30 results from inhibition of mTOR-mediated phosphorylation of S473, rather than from inhibition of an off-target kinase, or from an effect of mTOR inhibition unrelated to S473-P. To do this, we examined the effect of PP242 on T308 phosphorylation in two situations in which Akt could not be phosphorylated on S473.

**Figure 3:**
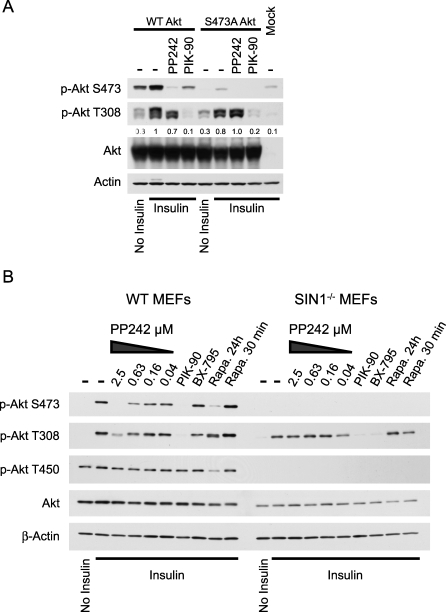
PP242 Does Not Directly Inhibit Phosphorylation of Akt at T308 (A) pT308 is not inhibited by PP242 in cells overexpressing S473A Akt. HEK293 cells were transfected with wild-type Akt, S473A Akt, or not transfected (Mock) and were treated with 2.5 μM PP242 or 625 nM PIK-90 as indicated prior to insulin stimulation. Lysates were analyzed by Western blotting. Quantitation of pT308 relative to insulin treated cells overexpressing wild-type Akt (lane 2) is shown below that blot. Data are representative of two independent experiments. (B) pT308 is not inhibited by PP242 in SIN1^−/−^ MEFs, which lack pS473. Primary wild-type (WT) and SIN1^−/−^ MEFs were pre-treated with 625 nM PIK-90, 10 μM BX-795, or 100 nM rapamycin for 24 h, 100 nM rapamycin for 30 min, or the indicated concentrations of PP242 prior to stimulation with insulin. Lysates were analyzed by Western blotting.

First, we overexpressed S473A mutant Akt and stimulated these cells with insulin (Figure 3A). S473A Akt was phosphorylated on T308 to a similar level as wild-type, yet in contrast to the wild-type, T308-P on S473A Akt was not inhibited by PP242. The lack of effect of PP242 on S473A Akt confirms that PP242 inhibition of pT308 requires S473 and also that PP242 does not inhibit PDK1 in cells, as was suggested by direct testing of PDK1 in vitro (Figure 1).

As a further test of the specificity of PP242 and the requirement for functional S473 phosphorylation in order for PP242 to inhibit T308-P, we examined the effect of PP242 on the phosphorylation of Akt in primary MEFs from embryos that lack SIN1 [16] (Figure 3B). SIN1 is a component of mTORC2, and knockout of SIN1 compromises the physical integrity of mTORC2 leading to a complete loss of Akt phosphorylation at S473 without affecting its phosphorylation at T308. Consistent with our results from L6 cells, PP242 inhibited the phosphorylation of Akt at both S473 and T308 in wild-type MEFs. By contrast, PP242 had no effect on the phosphorylation of T308 in SIN1^−/−^ MEFs that lack mTORC2. Furthermore, PP242 had no effect on the constitutive phosphorylation of the turn motif of Akt at T450 [16,31]. As a further comparison, we examined the effect of long-term rapamycin, which is known to block the assembly of mTORC2 is some cell lines [18]. Similar to PP242, long-term rapamycin treatment of wild-type MEFs inhibited S473-P and reduced the phosphorylation of T308-P, as was seen previously [18]. Importantly, the PI3K inhibitor PIK-90 and the PDK1 inhibitor BX-795 [32] blocked phosphorylation of T308 in SIN1^−/−^ MEFs, indicating that the failure of PP242 to block T308 in SIN1^−/−^ MEFs does not reflect a general resistance of T308 to dephosphorylation in cells that lack mTORC2. From these data, we conclude that PP242′s effect on T308-P is dependent on its inhibition of Akt phosphorylation by mTOR at S473. It remains unclear why mTORC2 knockout cells, but not cells treated with RNAi or pharmacological inhibitors of mTORC2, are able to retain T308 phosphorylation in the absence of phosphorylation at S473. However, there are a growing number of examples in which genetic deletion of a kinase results in compensatory changes that mask relevant phenotypes observed with the corresponding small molecule inhibitor [33].

### Akt Substrate Phosphorylation Is Only Modestly Inhibited by PP242

**Figure 4:**
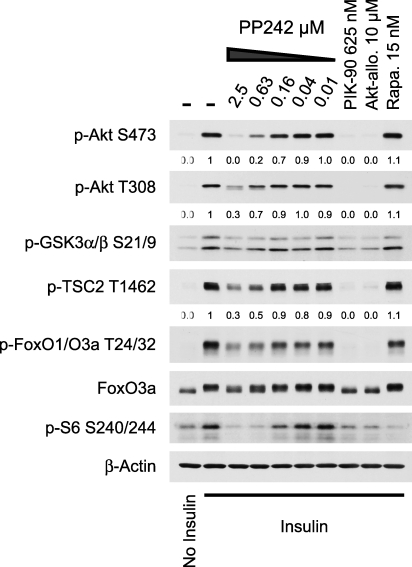
Phosphorylation of the Akt Substrates GSK3α/β, TSC2, and FoxO1/O3a Is Not Potently Inhibited by PP242 Lysates from L6 myotubes treated with kinase inhibitors and stimulated with insulin were analyzed by Western blotting. Quantitation of pAkt and pTSC2 relative to the insulin control (lane 2) is show below these blots.

Akt requires phosphorylation at both S473 and T308 for full biochemical activity in vitro [13], but it is unclear whether all of the cellular functions of Akt require it to be dually phosphorylated. Singly phosphorylated (T308-P) Akt from SIN1^−/−^ MEFs is competent to phosphorylate the cytoplasmic Akt substrates GSK3 and TSC2, but not the nuclear target FoxO [16]. Because low concentrations of PP242 inhibit the phosphorylation of S473 and higher concentrations partially inhibit T308-P in addition to S473-P, we used PP242 to examine whether some substrates of Akt are especially sensitive to loss of S473-P (Figure 4). We compared PP242 to the PI3K inhibitor PIK-90 and the allosteric Akt inhibitor Akti-1/2 [34], which inhibit the phosphorylation of Akt at both sites. In contrast to PIK-90 and Akti-1/2, which completely inhibited the phosphorylation of Akt and its direct substrates, PP242 only partially inhibited the phosphorylation of cytoplasmic and nuclear substrates of Akt. This suggests that phosphorylation of the Akt substrates we examined is only modestly sensitive to loss of S473-P. A caveat of comparing Akt substrates in Sin1^−/−^ MEFs with PP242-treated cells is the different turn motif (T450-P) status in these two conditions (Figure 3B).

In contrast to Akt, which maintains T308-P, SGK activity is completely inhibited by genetic disruption of mTORC2 [7]. Because SGK can phosphorylate FoxO and its activity is completely inhibited by disruption of mTORC2, it was suggested that the loss of FoxO phosphorylation in SIN1^−/−^ MEFs indicates that FoxO is primarily phosphorylated by SGK rather than Akt [7]. Because Akti-1/2 does not inhibit SGK [34] but inhibits FoxO1/O3a phosphorylation at T24/T32 in L6 myotubes (Figure 4), our data suggests that the major kinase for T24/T32 of FoxO1/O3a in L6 myotubes is Akt and not SGK.

### PP242 Does Not Have an Obvious Effect on Actin Stress Fibers

**Figure 5:**
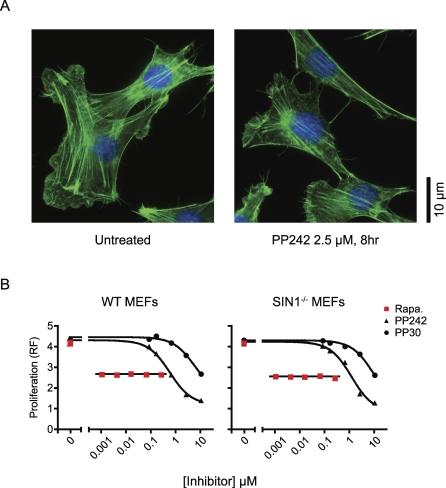
PP242 Inhibits Proliferation without Affecting Actin Stress Fibers (A) NIH 3T3 cells were stained for actin with Alexa 488-phalloidin (green) and for DNA with DAPI (blue). Images are representative of greater that 100 cells. (B) Differential inhibition of cell proliferation by PP242 and rapamycin does not require mTORC2. Proliferation of primary MEFs cultured for 3 d in the presence of kinase inhibitors was assayed by resazurin fluorescence (RF) and is shown in arbitrary units.

TORC2 is required for the generation of a polarized actin cytoskeleton in yeast [35]. Previous analysis of mTORC2 function using RNAi revealed a role for mTORC2 in the control of the actin cytoskeleton [3,4], yet these findings were not confirmed in primary MEFs lacking mTORC2 [15,17]. We examined actin stress fibers in NIH 3T3 cells (Figure 5) and in primary MEFs (unpublished data) treated with PP242. After 8 h of treatment with PP242, we found no obvious effect on the morphology or abundance of actin stress fibers (Figure 5), suggesting that mTORC2 activity is not required for the maintenance of actin stress fibers in these cells. That PP242 didn't obviously affect the morphology or abundance of actin stress fibers, does not rule out a role for mTOR in the control of the actin cytoskeleton, but it does show that pharmacological inhibition of mTORC2 does not affect the obvious changes in actin structure seen with RNAi.

### PP242 Inhibits Proliferation More Completely Than Rapamycin

We next measured the effect of dual mTORC1/mTORC2 inhibition by PP242 on the proliferation of primary MEFs (Figure 5B). For this analysis, we compared PP242 to selective mTORC1 inhibition by rapamycin. Rapamycin was tested at concentrations above its mTOR IC_50_, and at all concentrations tested, it inhibited growth to the same extent. By contrast, PP242 had a dose-dependent effect on proliferation and at higher doses was much more effective than rapamycin at blocking cell proliferation. The ability of PP242 to block cell proliferation more efficiently than rapamycin could be a result of its ability to inhibit mTORC1 and mTORC2, because rapamycin can only inhibit mTORC1. To test this possibility, we measured the effects of both compounds on the proliferation of SIN1^−/−^ MEFs, which lack mTORC2. In SIN1^−/−^ MEFs, rapamycin was also less effective at blocking cell proliferation than PP242. That PP242 and rapamycin exhibit very different anti-proliferative effects in SIN1^−/−^ MEFs suggests that the two compounds differentially affect mTORC1.

### Rapamycin-Resistant mTORC1

**Figure 6:**
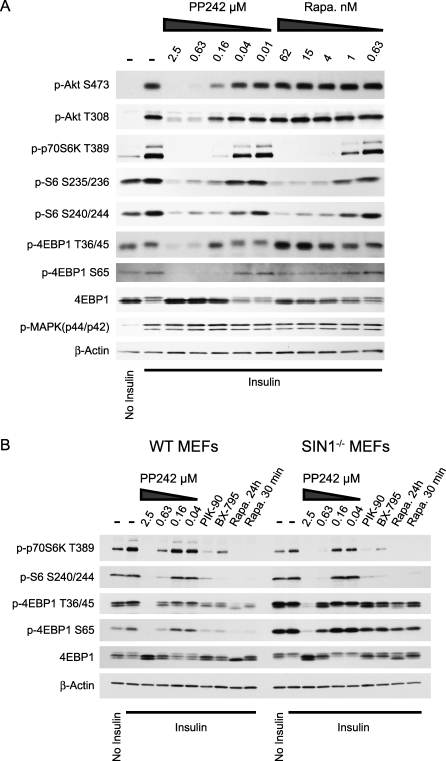
PP242 Inhibits Rapamycin-Resistant mTORC1 (A) Western blots of lysates from L6 myotubes treated with kinase inhibitors and stimulated with insulin for 10 min. (B) Western blots of lysates from Figure 3B. Actin loading control is repeated here for clarity.

mTORC1 regulates protein synthesis by phosphorylating the hydrophobic motif of p70S6-Kinase (S6K) at T389 and the eIF4E-binding-protein, 4EBP1, at multiple sites. Our proliferation experiments suggest that rapamycin and PP242 have distinct effects on mTORC1. We compared the effects of acute treatment with rapamycin and PP242 on S6K, ribosomal protein S6 (S6), and 4EBP1 phosphorylation (Figure 6A) to see if these inhibitors differentially affect the phosphorylation of these canonical substrates of mTORC1. Both rapamycin and PP242 inhibited the phosphorylation of S6K and its substrate S6, and neither rapamycin nor PP242 affected the phosphorylation of 4EBP1 on T70 (Figure S2A). In contrast, PP242 fully inhibited the phosphorylation of 4EBP1 at T36/45 and S65, whereas rapamycin only had a modest affect on these same phosphorylations. Treatment of cells with PP30 was also effective at reducing the phosphorylation of 4EBP1 at T36/45 (Figure S3), indicating that the block of T36/45 phosphorylation by PP242 is due to its inhibition of mTOR and not PKC-alpha. PIK-90 did not reduce the phosphorylation of 4EBP1 at T36/45, demonstrating that inhibition of PI3K and Akt activation alone is not sufficient to block the phosphorylation of 4EBP1 at T36/45 (Figure S3).

The enhanced dephosphorylation of 4EBP1 caused by PP242 as compared with rapamycin could be due to incomplete inhibition of mTORC1 by rapamycin or involvement of mTORC2 in the phosphorylation of 4EBP1. To examine these alternatives, we analyzed the effect of PP242 and rapamycin on the phosphorylation of 4EBP1 in SIN1^−/−^ MEFs that lack mTORC2 (Figure 6B). SIN1^−/−^ MEFS showed higher levels of p4EBP1, suggesting that due to the lack of mTORC2, these cells have more mTORC1 activity, although stronger S6K phosphorylation in wild-type cells contradicts this simple interpretation. Despite an increase in p4EBP1 in SIN1^−/−^ compared with wild-type MEFs, shorter exposures of the p4EBP1 blots (Figure S2B) show that PP242 inhibits p4EBP1 with the same potency in both cells. The fuller inhibition of p4EBP1 by PP242 than by rapamycin in wild-type and SIN1^−/−^ MEFs indicates that the presence of mTORC2 is not required for rapamycin and PP242 to have distinct effects on 4EBP1 phosphorylation, and suggests that PP242 is a more complete inhibitor of mTORC1 than rapamycin.

### Inhibition of Translation by TORKinibs

While the precise role of S6K in translation control is still poorly understood, it is known that the hypophosphorylated 4EBP1 protein acts as negative regulator of the major cap-binding protein eIF4E. We directly assessed the effect of PP242 on cap-dependent translation downstream of mTOR activation. The phosphorylation of 4EBP1 by mTOR in response to growth factor and nutrient status causes it to dissociate from eIF4E allowing eIF4G and associated factors to bind to the 5' cap, recruit the 40S subunit of the ribosome, and scan the mRNA for the start codon to initiate translation. The phosphorylation of 4EBP1 by mTOR is complicated in that it occurs at multiple sites, and not all sites are equally effective at causing dissociation of 4EBP1 from eIF4E [36]. Furthermore, a hierarchy is thought to exist whereby the N-terminal threonine phosphorylations at 36/45 precede and are required for the C-terminal phosphorylations at S65 and T70 [37,38]. Phosphorylation at S65 causes the greatest decrease in affinity of 4EBP1 for eIF4E [39,40], and S65 is probably the most important site in cells for dissociation of 4EBP1 from eIF4E [41], but other sites are also important [36,42].

**Figure 7:**
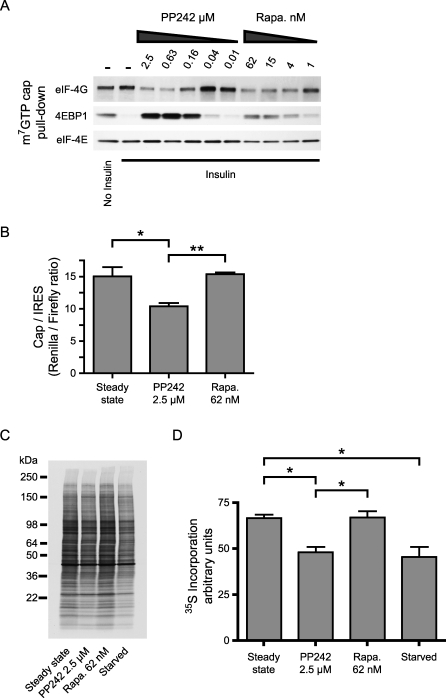
PP242 Inhibits Cap-Dependent Translation (A) Cap-binding proteins in lysates from Figure 6A were purified by 7-methyl GTP (m^7^GTP) affinity and analyzed by Western blotting. (B) Primary MEFs were transfected with a bicistronic reporter vector. The ratio of renilla (cap-dependent) to firefly (IRES-dependent) luciferase activity was measured after incubation overnight in either 10% serum (steady state) or with the indicated inhibitors in the presence of 10% serum (*n* = 3). *p < 0.05, **p < 0.01, ANOVA with Tukey's post test. (C) Primary MEFs were incubated overnight as in (B) prior to labeling new protein synthesis with ^35^S. Newly synthesized proteins were separated by SDS-PAGE, transferred to nitrocellulose and visualized by autoradiography. (D) Newly synthesized protein from three experiments as in (C) was quantified. *p < 0.05, ANOVA with Tukey's post test.

We examined the effect of PP242 on the active eIF4E initiation complex of translation by using a cap-binding assay. eIF4E binds tightly to beads coated with the cap analogue 7-methyl GTP (m^7^GTP), allowing proteins bound to eIF4E to be examined. Rapamycin caused partial inhibition of the insulin-stimulated release of 4EPB1 from eIF4E (Figure 7A), consistent with its partial inhibition of S65 phosphorylation (Figure 6A). The rapamycin-induced retention of 4EBP1 was accompanied by a loss of recovery of eIF4G, because the binding of 4EBP1 and eIF4G to eIF4E are mutually exclusive. In contrast, treatment with PP242 caused a much larger retention of 4EBP1, raising the retention of 4EBP1 above the level seen in unstimulated serum-starved cells, which are known to have low levels of protein translation [43].

Translation initiation depending on eIF4E activity is the rate-limiting step in cap-dependent protein translation [44]. PP242 caused a higher level of binding between 4EBP1 and eIF4E than rapamycin (Figure 6A), suggesting that cap-dependent translation will be more highly suppressed by PP242 than by rapamycin. To quantify the efficiency of cap-dependent translation in the presence of PP242 and rapamycin, we used the well-established bicistronic reporter assay where translation initiation of the first cistron is dependent on the 5′ cap, whereas initiation of the second cistron depends on a viral internal ribosome entry site (IRES) that bypasses the need for cap-binding proteins such as eIF4E [45]. PP242 caused a significant decrease in cap-dependent, but not IRES-dependent (Figure S4), translation, whereas rapamycin did not have a statistically significant effect on cap-dependent translation (Figure 7B), consistent with the modest effect of rapamycin on 4EBP1 phosphorylation (Figure 6A). Based on this assay, inhibition of mTOR and p4EBP1 reduces cap-dependent translation by about 30%, suggesting that cap-dependent translation is only partially inhibited by hypophosphorylated 4EBP1. The majority of protein synthesis is thought to be cap-dependent [44], and consistent with this we find that PP242 also reduces total protein synthesis by about 30%, whereas rapamycin does not have a significant effect (Figure 7C and 7D).

### Inhibition of mTORC1 and mTORC2 In Vivo

**Figure 8:**
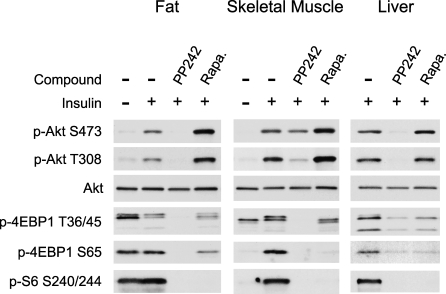
PP242 Inhibits mTORC2 and Rapamycin-Resistant mTORC1 In Vivo Rapamycin (5 mg/kg), PP242 (20 mg/kg), or vehicle were injected into the intraperitoneal (IP) cavity of mice, followed by IP injection of 250 mU insulin or saline. Lysates were prepared from perigenital fat, leg muscle, and liver and analyzed by Western blotting.

Mouse knock-outs of mTORC1 or mTORC2 result in embryonic lethality and thus it has been difficult to examine the effects of loss of mTOR in animals. To begin to explore the tissue specific roles of mTORC1 and mTORC2 and confirm the pathway analysis from cell culture experiments, we treated mice with PP242 and rapamycin and examined the acute effect of these drugs on insulin signaling in fat, skeletal muscle, and liver tissue (Figure 8). In fat and liver, PP242 was able to completely inhibit the phosphorylation of Akt at S473 and T308, consistent with its effect on these phosphorylation sites observed in cell culture. Surprisingly, PP242 was only partially able to inhibit the phosphorylation of Akt in skeletal muscle and was more effective at inhibiting the phosphorylation of T308 than S473, despite it's ability to fully inhibit the phosphorylation of 4EBP1 and S6. These results will be confirmed by in vivo dose-response experiments, but, consistent with the partial effect of PP242 on pAkt in skeletal muscle, a muscle-specific knockout of the integral mTORC2 component rictor resulted in only a partial loss of Akt phosphorylation at S473 [46]. These results suggest that a kinase other than mTOR, such as DNA-PK [8,9], may contribute to phosphorylation of Akt in muscle.

Rapamycin often stimulates the phosphorylation of Akt [47,48], probably by relieving feedback inhibition from S6K to the insulin receptor substrate 1 (IRS1) [49], a key signaling molecule that links activation of the insulin receptor to PI3K activation. In all tissues examined, and especially in fat and muscle, acute rapamycin treatment activated the phosphorylation of Akt at S473 and T308 (Figure 8). In contrast to rapamycin, by inhibiting both mTORC2 and mTORC1, PP242 suppresses rather than enhances Akt activation.

As was seen in cell culture, rapamycin and PP242 also differentially affect the mTORC1 substrates S6K and 4EBP1 in vivo. S6 phosphorylation was fully inhibited by rapamycin and PP242 in all tissues examined. While PP242 was effective at blocking the phosphorylation of 4EBP1 on both T36/45 and S65 in all tissues examined, rapamycin did not block 4EBP1 phosphorylation as completely as PP242. Further experiments will be required to identify the mechanism by which 4EBP1 phosphorylation is partially resistant to rapamycin.

## Discussion

Rapamycin has been a powerful pharmacological tool allowing the discovery of mTOR's role in the control of protein synthesis. Since the discovery of a rapamycin-insensitive mTOR complex, there has been a significant effort to develop pharmacological tools for studying this complex. We have used two structurally distinct compounds to pharmacologically dissect the effects of mTOR kinase inhibition toward mTORC1 and mTORC2 activity.

We have shown through the use of these inhibitors that the inhibition of mTOR kinase activity is sufficient to prevent the phosphorylation of Akt at S473, providing further evidence that mTORC2 is the kinase responsible for Akt hydrophobic motif phosphorylation upon insulin stimulation. We also find that phosphorylation at T308 is linked to phosphorylation at S473, as had been observed in experiments where mTORC2 was disabled by RNAi and long-term rapamycin, but not homologous recombination. Surprisingly however, inhibition of mTORC2 does not result in a complete block of Akt signaling, as T308P is partially maintained and Akt substrate phosphorylation is only modestly affected when S473 is not phosphorylated.

Despite its modest effect on Akt substrate phosphorylation, PP242 was a strikingly more effective anti-proliferative agent than rapamycin. These results were reproduced even in cells lacking mTORC2 (SIN1^−/−^), suggesting that downstream mTORC1 substrates might be responsible for PP242′s strong anti-proliferative effects. Interestingly, we observe that phosphorylation of the mTORC1 substrate 4EBP1 is partially resistant to rapamycin treatment at concentrations that fully inhibit S6K, whereas PP242 completely inhibits both S6K and 4EBP1. Because rapamycin can only partially inhibit the phosphorylation of 4EBP1, but it can fully in inhibit the phosphorylation of S6K, rapamycin appears to be a substrate-selective inhibitor of mTORC1. Consistent with this finding, experiments with purified proteins have shown that rapamycin/FKBP12 only partially inhibits the in vitro phosphorylation of 4EBP1 at Ser 65 by mTOR but can fully inhibit the in vitro phosphorylation of S6K [50]. By contrast, LY294002, a direct inhibitor of many PI3K family members including mTOR, was equally effective at inhibiting the phosphorylation of S6K and 4EBP1 by mTOR in vitro [50] and in cells [23], although this finding is complicated by LY294002′s inhibition of multiple lipid and protein kinases [51] including PIM, a kinase potentially upstream of 4EBP1 phosphorylation [52,53]. These results argue that PP242, in addition to being useful for investigating mTORC2, can reveal rapamycin-resistant components of mTORC1 function. Indeed, proliferation of SIN1^−/−^ MEFs is more sensitive to PP242 than rapamycin (Figure 5B), suggesting that rapamycin-resistant functions of mTORC1, including the aspects of translation initiation highlighted in Figure 7, are key to the anti-proliferative effects of PP242. Furthermore, our findings suggest that the inhibition of translational control and the anti-proliferative effects of PP242 require inhibition of 4EBP1 phosphorylation and eIF4E activity.

Using TORKinibs to acutely inhibit mTOR has surprisingly led to the identification of outputs from mTORC1 that are rapamycin-resistant. These observations should motivate further studies aimed at understanding how rapamycin is able to selectively affect different outputs downstream of mTORC1. As active site inhibitors of mTOR join rapamycin and its analogs in the clinic [22,27,30], it will be important to understand the distinct effects of these pharmacological agents on cellular and organismal physiology and to evaluate their efficacy in the treatment of disease and cancer caused by hyperactivation of the PI3K→Akt→TOR pathway.

## Materials and Methods

### Ethics statement.

Mice were handled in accordance with protocols approved by the committee for animal research at the University of California San Francisco, United States of America.

### Cell culture.

Cells were grown in DMEM supplemented with 10% FBS, glutamine, and penicillin/streptomycin. Confluent L6 myoblasts were differentiated into myotubes by culturing them for 5 d in medium containing 2% FBS. L6 myotubes were maintained in medium containing 2% FBS until use. Primary wild-type MEFs used in Figure 7 were isolated at embryonic day 13.5 as previously described [54]. Primary SIN1^−/−^ MEFs and matching wild-type controls were provided by B. Su and isolated as previously described [16].

### Cell lysis and Western blotting.

Except where indicated otherwise, cells were serum starved overnight and incubated with inhibitors or 0.1% DMSO for 30 min prior to stimulation with 100 nM insulin for 10 min. All inhibitors were either synthesized as previously described [21,24,55] or were from Calbiochem (rapamycin and Akti-1/2). Cells were lysed by scraping into ice cold lysis buffer followed by brief sonication. Lysates were cleared by centrifugation, resolved by SDS-PAGE, transferred to nitrocellulose, and immunoblotted with antibodies from Cell Signaling Technology. Unless otherwise indicated, cells were lysed in 300 mM NaCl, 50 mM Tris pH 7.5, 5 mM EDTA, 1% Triton X-100, 0.02% NaN_3_, 20 nM microcystin (Calbiochem), Sigma phosphatase inhibitor cocktails 1 and 2, Roche protease inhibitor cocktail, and 2 mM PMSF. For Figures 6A and 7A, and Figure S2A, cells were lysed in cap lysis buffer (140 mM KCl, 10 mM Tris pH 7.5, 1 mM EDTA, 4 mM MgCl_2_, 1 mM DTT, 1% NP-40, 20 nM microcystin, Sigma phosphatase inhibitor cocktails 1 and 2, Roche protease inhibitor cocktail without EDTA and 2 mM PMSF).

### Cap pull-down assay.

L6 myotubes from one well of a six-well plate were lysed in 300 μl of cap lysis buffer as described above. 50 μl of detergent-free cap lysis buffer and 20 μl of pre-washed cap beads were added to 150 μl of cleared lysate and incubated at 4 °C overnight with tumbling. The beads were washed twice with 400 μl of cap wash buffer (cap lysis buffer with 0.5% NP-40 instead of 1% NP-40) and twice with 500 μl of PBS. The beads were boiled in SDS-PAGE sample buffer and the retained proteins analyzed by Western blot. All antibodies were from Cell Signaling Technologies except for the anti-eIF4E antibody, which was from BD Biosciences.

### Kinase assays.

Phosphorylation of histone H1 (4 μM) by PKC was assayed in a buffer containing 200 ng/ml recombinant kinase, 25 mM HEPES pH 7.5, 10 mM MgCl_2_, 5 mM ß-glycerol phosphate, 0.05 mg/ml phosphatidylserine, 0.03% Triton X-100, 0.5 mg/ml BSA, 2.5 mM DTT, 100 μM CaCl_2_, 1 μM PMA, 10 μM ATP, and 15 μCi/ml of γ-^32^P-ATP. Inhibitors were tested in a four-fold dilution series from 10 μM to 600 pM, and four measurements were made at each concentration. The kinase reaction was terminated by spotting onto nitrocellulose, which was washed 5 times with 1 M NaCl/1% phosphoric acid. The radioactivity remaining on the nitrocellulose sheet was quantified by phosphorimaging, and IC_50_ values were determined by fitting the data to a sigmoidal dose-response curve using the Prism software package.

PDK1, mTORC1, and mTORC2 were assayed as previously described [21].

### In-cell Western.

L6 myotubes were grown and differentiated in 96-well plates. The outside wells of the plate were not used for the experiment, but were kept filled with media. Following stimulation, cells were fixed for 15 min with 4% formaldehyde in PBS with Ca^++^ and Mg^++^. The cells were washed three times with PBS and the blocked and permeabilized with 5% goat serum in PBS with 0.3% Triton X-100 (PBS-GS-TX). Primary antibodies to S473 (Cell Signaling #4060) and T308 (Cell Signaling #2965) were added at 1:1000 and 1:500, respectively, in PBS-GS-TX, and the plates were incubated at 4 °C overnight. The plates were then washed three times with PBS, and goat anti-rabbit secondary antibody (Pierce Biotechnology) was added at 0.01 μg/ml in PBS-GS-TX. After 1 h at room temperature, plates were washed three times with PBS. ELISA chemiluminescent reagent (Femto, Pierce Biotechnology) was added to each well and after 1 min, the plate was read in a luminescence plate reader using a 100-ms integration time. The pAkt signal from pT308 and pS473 was normalized to control wells, so that 0 represents the level of pAkt in serum starved cells and 1 represents the level upon insulin stimulation. EC_50_ values were determined by fitting the data to a sigmoidal dose-response curve using the Prism software package. The significance of differences between EC_50_ values was evaluated using the F test.

### Akt transfection.

Akt was transfected into HEK293 cells using Lipofectamine 2000 according the manufacturers protocol. Two days after transfection, cells were serum starved overnight and the next day they were treated with inhibitors and processed for western blotting as described above.

### Actin cytoskeleton staining.

NIH 3T3 cells were plated on poly-lysine coated coverslips at 30% confluence the day before the experiment. Following treatment with PP242 or 0.1% DMSO for 8 h in 10% serum growth medium, the actin cytoskeleton was stained as previously described [24].

### Bicistronic reporter assay.

Primary MEFs were transfected with a bicistronic reporter [54] containing a viral IRES using Lipofectamine 2000 according to the manufacturers protocol. At 2 d post transfection, cells were treated overnight with compounds as indicated or starved of serum. The next day, Renilla and Firefly luciferase activity were measured using the Dual-Luciferase kit (Promega). Differences in the ratio of Renilla to Firefly luciferase signals were analyzed for statistical significance by one-way ANOVA with Tukey's post test using the Prism software package.

### ^35^S labeling of new protein synthesis.

Primary MEFs grown to 70% confluence in six-well plates were incubated overnight in either 10% Serum (Steady State), kinase inhibitors in 10% serum, or 0.1% serum (starved). Cells were then washed once with DMEM lacking cysteine and methionine (DMEM-noS), and the medium was replaced with DMEM-noS including dialyzed serum and kinase inhibitors as indicated. After incubation for 1 h, 50 μCi of Expre^35^S^35^S (NEN) was added to each well and the cells were labeled for 4 h. Cells were washed once with ice-cold PBS, and lysed as described above for Western blotting. Following separation by SDS-PAGE, and transfer to nitrocellulose, ^35^S-labeled proteins were visualized by autoradiography with film. For quantitation, the membrane was exposed to a phosphorimager screen and the resulting image was quantified in ImageJ. Differences in ^35^S incorporation were analyzed for statistical significance by one-way ANOVA with Tukey's post test using the Prism software package.

### In vivo drug treatment and Western blotting.

Drugs were prepared in 100 μl of vehicle containing 20% DMSO, 40% PEG-400, and 40% saline. Six-wk-old male C57BL/6 mice were fasted overnight prior to drug treatment. PP242 (0.4 mg), rapamycin (0.1 mg), or vehicle alone was injected IP. After 30 min for the rapamycin-treated mouse or 10 min for the PP242 and vehicle-treated mice, 250 mU of insulin in 100 μl of saline was injected IP. 15 min after the insulin injection, the mice were killed by CO_2_ asphyxiation followed by cervical dislocation. Tissues were harvested and frozen on liquid nitrogen in 200 μl of cap lysis buffer. The frozen tissue was thawed on ice, manually disrupted with a mortar and pestle, and then further processed with a micro tissue-homogenizer (Fisher PowerGen 125 with Omni-Tip probe). Protein concentration of the cleared lysate was measured by Bradford assay and 5–10 μg of protein was analyzed by Western blot as described above.

### Cell proliferation assay.

Wild-type and SIN1^−/−^ MEFs were plated in 96-well plates at approximately 30% confluence and left overnight to adhere. The following day cells were treated with PP242, rapamycin, or vehicle (0.1% DMSO). After 72 h of treatment, 10 μl of 440 μM resazurin sodium salt (Sigma) was added to each well, and after 18 h, the florescence intensity in each well was measured using a top-reading florescent plate reader with excitation at 530 nm and emission at 590 nm.

## Supporting Information

Figure S1 PP242 Inhibits Akt Phosphorylation over the Course of 1 h L6 myotubes were pre-treated with PP242 or DMSO for 30 min and stimulated with insulin for the indicated times prior to lysis and analysis by Western blotting.
(4.31 MB AI).

Figure S2 Additional Analysis of 4EBP1 Phosphorylation (A) 4EBP1 phosphorylation at T70 is not inhibited by either PP242 or rapamycin. L6 myotube lysates from Figure 6A were analyzed by Western blotting.
(B) 4EBP1 phosphorylation is inhibited by PP242 with similar potency in SIN1^−/−^ and wild-type (WT) MEFs. Western blotting from Figure 6B is shown with shorter exposures of p4EBP1.
(4.97 MB AI).

Figure S3 Rapamycin-Resistant Phosphorylation of 4EBP1 Is Sensitive to the TORKinibs PP30 and PP242, but Not the PI3K Inhibitor PIK-90 L6 myotube lysates from Figure 2A were analyzed by Western blotting.
(2.44 MB AI).

Figure S4 PP242 Inhibits Cap, but Not IRES-Dependent, Translation (A) Renilla (cap-dependent) luciferase activity from samples in Figure 7B.
(B) Firefly (IRES-dependent) luciferase activity from samples in Figure 7B. Firefly luciferase activity of the PP242 treated sample is not significantly different from control (*p* = 0.4, ANOVA).
(1.55 MB AI).

Table S1 In Vitro IC_50_ Determinations Using Three Forms of mTOR (19 KB DOC)

## Abbreviations

IRES: internal ribosome entry site
MEF: mouse embryonic fibroblast
mTOR: mammalian target of rapamycin
PI3K: phosphoinositide 3-kinase
RNAi: RNA interference
TORKinibs: TOR kinase domain inhibitors

## Note Added in Proof

Because reference 56 was added during the proof stage, it is cited out of order.
