# Supplementary material for: Active-Site Inhibitors of mTOR Target Rapamycin-Resistant Outputs of mTORC1 and mTORC2
Source: PLoS Biol. 2009 Feb 10;7(2):e1000038. doi: 10.1371/journal.pbio.1000038 (PMC2637922; doi:10.1371/journal.pbio.1000038)
Supplement: Supplementary file 1 [file pbio.1000038.st001.doc]

In Vitro IC_50_ Determinations Using Three Forms of mTOR

IC_50_ µM

mTOR (Invitrogen)	mTORC1	mTORC2

PP242	0.008	0.030	0.058

PP30	0.080	0.128	0.281

[ATP] µM	10	100	100
